# Advances in Crohn's Disease: A Comprehensive Review of Pathophysiology, Diagnosis, and Emerging Therapeutics

**DOI:** 10.1002/jgh3.70394

**Published:** 2026-04-05

**Authors:** Yoon‐Kyo An, Robert Gilmore, Graham Radford‐Smith, Jakob Begun

**Affiliations:** ^1^ Department of Gastroenterology and Hepatology Mater Hospital Brisbane South Brisbane Queensland Australia; ^2^ Inflammatory Bowel Disease Research Group Mater Research Institute‐UQ South Brisbane Queensland Australia; ^3^ Faculty of Medicine The University of Queensland Herston Queensland Australia; ^4^ Gut Health Research Group QIMR Berghofer Medical Research Institute Herston Queensland Australia; ^5^ Department of Gastroenterology and Hepatology Royal Brisbane and Women's Hospital Herston Queensland Australia

**Keywords:** Crohn's disease, epidemiology, inflammatory bowel disease, management, pathophysiology

## Abstract

Crohn's disease (CD) is a chronic inflammatory condition of the bowel with a rising global incidence. Its complex etiology involves genetic susceptibility, environmental factors, and alterations in the gut microbiota, leading to dysregulated immune responses. Prompt diagnosis and personalized treatment are essential to prevent disease flares and complications. This review provides a comprehensive overview of CD, including its epidemiology, pathophysiology, diagnosis, management, treatment goals, and prospects. Treatment goals in CD have evolved beyond symptomatic control to achieving mucosal healing to prevent disease related complications. Advances in biologic therapies including the use of anti‐TNF agents, vedolizumab, and ustekinumab in CD have improved outcomes, but challenges such as immunogenicity and accessibility remain. A ‘treat‐to‐target’ approach based on biomarkers such as C‐reactive protein and fecal calprotectin allows for objective disease monitoring and adjustment of treatment as needed to achieve these targets. Promising new drug classes including Janus kinase inhibitors and anti‐interleukin‐23 antibodies are emerging as potential therapies. Personalized medicine is evolving as a vital approach to tailor treatment based on individual genomic, clinical, and environmental factors. Future research to refine CD management should focus on predictive biomarkers, treatment algorithms, and head‐to‐head trials. Overall to improve outcomes and quality of life for those living with CD, management requires a multifaceted approach that considers disease severity, individualized patient characteristics, and the evolving therapeutic landscape.

## 1 Introduction

Crohn's disease (CD) is a chronic inflammatory condition that affects the gastrointestinal tract, and its incidence is on the rise globally. This complex disorder can arise from a combination of genetic susceptibility, environmental influences, and changes in the gut microbiota, leading to dysregulated immune responses. The onset of the disease at a young age in most cases necessitates prompt together with long‐term treatment to prevent disease flares and disease progression with intestinal complications. The critical evaluation of disease extent and other prognostic factors such as disease behavior is essential to inform treatment choices. Treatment goals in CD have evolved beyond symptomatic control to achieve mucosal healing to prevent complications and surgery. These approaches include early immunosuppression or combination therapy with biologics in high‐risk patients, coupled with vigilant monitoring and therapy adjustment based on the “treat‐to‐target” strategy. As the therapeutic options for CD expand, the development of biomarkers to predict and monitor treatment response is becoming increasingly crucial for personalized medical decision making in the future. This summary will provide an overview of CD in adults, covering its epidemiology, pathophysiology, diagnosis, extraintestinal manifestations, medical management, treatment goals, disease monitoring, impact on quality of life and outlook for patients. It differs from past reviews in the discussion of novel therapies, novel diagnostic and monitoring strategies and inclusion of dietary measures [1, 2, 3].

## 2 Epidemiology

### 2.1 Incidence and Prevalence of CD

Inflammatory bowel diseases (IBD), including CD and ulcerative colitis (UC), are chronic inflammatory disorders affecting the gastrointestinal tract and characterized by a progressive and unpredictable disease course. There has been a global rise in the incidence of overall IBD during the last few decades [1]. CD can develop at any age, but the typical onset is between the second and fourth decades of life, with a smaller peak reported between the ages of 50–60 years [4]. In general, CD affects men and women equally [5] however the recent study showed the differences in gender‐specific incidence, with a female predominance in CD in western Caucasian populations and a male predominance in the Asian population [6].

Over time, CD has shown a consistent and steady increase in incidence and prevalence in each country studied [1]. However, the incidence of CD varies significantly by region, with the highest reported rates in North America, northern and western Europe and Oceania [7]. The incidence ranges from 0.1 to 58 cases per 100 000 person‐years. Specifically, CD has an incidence of 0–20.2 cases per 100 000 person‐years in North America and 0.3–12.7 cases per 100 000 person‐years in Europe [7]. Comparatively, UC incidence rates are generally lower. Similarly, the prevalence of IBD is highest in Europe and North America, with Germany reporting 322 cases per 100 000 persons and Canada reporting 319 cases per 100 000 persons. In Australia, the incidence ranges from 24.2 to 32.2 cases per 100 000 person‐years [8] and a recent study showed that the estimated crude prevalence of IBD was 653 per 100 000 persons with 306 cases of CD in 100 000 persons and 334 cases of UC in 100 000 person [9].

### 2.2 Global Trends and Challenges

The turn of the century has witnessed a paradigm shift in CD epidemiology, with rapidly increasing incidence rates observed in newly industrialized countries in Asia, Africa, and South America [7]. The rapid changes in CD epidemiology are a global challenge for disease diagnosis, healthcare delivery, and disease prevention. Countries such as China, after undergoing urbanization, have seen a transformation from CD being a rare condition to becoming a substantial health burden. Furthermore, CD incidence in Asia has risen more rapidly than UC, although overall IBD prevalence remains lower than in Western nations [1].

Western lifestyle factors including diet, urbanization, and industrialization, is attributed to increased CD risk [10]. Migrants moving from low‐prevalence to high‐prevalence regions are at an increased risk of developing CD, with the risk being more pronounced in the offspring of immigrants [7].

### 2.3 Factors Contributing to CD Incidences and Progression

The development of CD is thought to arise from the complex interaction between genetic susceptibility, environmental factors, and the composition of the intestinal microflora. This interplay can lead to an abnormal mucosal immune response and impaired function of the epithelial barrier [5].

Genetic factors have a substantial impact on IBD risk, with familial inheritance recognized [11, 12]. Genome‐wide association studies (GWAS) have identified numerous genetic variants associated with CD risk, such as *NOD2*, *ATG16L1*, *IL23R* and *TNFSF15* [1, 13]. Of note, whereas non‐synonymous coding risk variants in *NOD2*, *ATG16L1* and *IL23R* predominate in Caucasian populations, the risk variants of these genes are monomorphic in Asian cohorts [5]. In Asians, *TNFSF15* is the predominant risk locus that is selectively associated with CD [14], and its effect size exceeds that seen with *NOD2* in Caucasian populations [15]. The varying effect of polymorphisms in these genes among different ethnicities, highlights the complexity of IBD genetics.

Environmental factors play a crucial role in the onset and progression of CD in genetically susceptible individuals [5, 7]. Smoking has been recognized as the sole modifiable risk factor for CD, contributing to a twofold increase in the likelihood of developing the condition and is associated with more severe, refractory disease [16]. Multiple studies have shown that current smokers were more likely to have perianal and stricturing disease than non‐smokers [17]. The impact of smoking on CD risk is more pronounced in female and may vary depending on age. Moreover, smoking is linked to early disease onset, a greater requirement for immunosuppression, an increased likelihood of surgical intervention, and higher rates of disease recurrence following surgery [5].

CD is characterized by gut dysbiosis, and among the various environmental factors that have changed in recent years, diet appears to have the most significant impact on the intestinal microbiota. Changes in food composition, such as a shift from high‐fiber, low fat foods to processed foods containing additives, have altered the host‐gut microbiota relationship. The reduction in dietary fiber intake and frequent fluctuation between high‐fiber and low‐fiber foods have resulted in decreased gut‐microbiota diversity, which is closely linked to the development of CD [18]. Furthermore, high sugar intake has been linked to an increased risk of developing IBD, through changes in the microbiome and alterations of epithelial metabolism [19]. Overall, epidemiological dietary studies point towards diets high in sugars and processed foods, as well as low fiber intake to increase the risk of developing IBD [20].

Urban living during childhood, oral contraceptives, aspirin, and NSAIDs have also been associated with an increased risk of CD [21]. Conversely, exposures to farm animals and pets during childhood, breastfeeding and statin use have been linked to decreased CD risk [1, 16]. Antibiotic exposure during the first year of life is associated with in an increased risk of IBD, likely through perturbations in the microbiome [22, 23]. While vitamin D deficiency is common among IBD patients, its direct role as risk factors for disease development remains unclear [24]. Thus, the epidemiologic research has identified numerous environmental exposures that appear to increase the risk of developing IBD through negative impacts on gut homeostasis leading to uncontrolled inflammation.

## 3 Pathophysiology

The complex interplay of impaired intestinal barrier function, dysregulated immune responses, and alterations in the gut microbiota can result in CD [25]. Understanding these mechanisms is crucial for developing targeted therapies and personalized treatment approaches for individuals living with CD.

### 3.1 Innate Immune Response

The innate immune system plays a significant role in CD pathogenesis. The intestinal barrier, consisting of intestinal epithelial cells (IECs), innate immune cells, intraepithelial lymphocytes (IELs), and the mucus layer, serve as the first line of defense against intestinal bacteria, pathogens, and food antigens [26]. The mucosal barrier function is largely influenced by IECs, and mutations in certain genes, such as *NOD2, ATG16L1, IRGM* and *LRRK2*, can result in abnormalities in the secretory activity of Paneth cells, which play a crucial role in gut homeostasis [27].

Intrinsic dysfunction of macrophages and neutrophil response to microbial stimuli can disrupt gut homeostasis. NOD‐like receptors initiate immune response to pathogen associated molecular patterns (PAMPs), and under these conditions dendritic cells become pro‐inflammatory and secrete protective cytokines [5]. Any defects in the above barrier components can lead to inflammation. Recently, the role of dietary emulsifiers in disruption of the intestinal barrier has been described [28]. Ingestion of the commonly used emulsifiers, namely carboxymethylcellulose and polysorbate‐80, induced inflammation in genetically susceptible mice and this was associated with a decrease in the thickness of the protective mucous layer and deep penetration of intestinal bacteria into the epithelial layer [28]. The importance of an intact barrier function is further supported by genetic susceptibility variants in genes responsible for junctional proteins, such as E‐cadherin, guanine nucleotide‐binding protein subunit‐α12 and zonula occluden 1 in IECs, along with impaired production of antimicrobial peptides by innate immune cells and IELs [29]. These functional alterations lead to increased intestinal permeability, a characteristic feature of CD. Specifically during active CD, there is reduced expression of the sealing tight junction proteins claudin 5 and claudin 8, and increased expression of pore‐forming claudin 5 in IECs [1]. Defects in tight junction proteins and endoplasmic reticulum (ER) stress in IECs have been associated with CD [30]. The role of ER stress is further highlighted by studies in mice that show a single point mutation in the MUC2 gene results in increased ER stress, impaired barrier function, and spontaneous colitis [31]. Impaired barrier function in CD contributes to abnormal innate immune responses, leading to chronic inflammation in the gastrointestinal tract triggered by dysregulated interaction with gut microbes.

### 3.2 Adaptive Immune Response

The adaptive immune response is a highly specialized and dynamic defense mechanism of the immune system that play a crucial role in CD by recognizing and targeting specific pathogens, but its dysregulation can lead to chronic inflammation and tissue damage in the gastrointestinal tract. CD is characterized by excessive T helper (Th) 1 and Th 17 cell responses in the setting of proinflammatory cytokines such as IL‐12, IL‐18 and IL‐23 [32]. These lymphocytes produce inflammatory cytokines IL‐17, IFNγ and TNF, perpetuating gut inflammation. Interestingly Th‐17 cells also produce IL‐22, which has a role in maintaining barrier function and driving wounding healing [33]. Impaired functional activity of intestinal regulatory T cells, which produce the anti‐inflammatory IL‐10 cytokine, has been reported in CD, leading to inadequate immune regulation [1]. Polymorphisms in genes encoding IL‐10 and the IL‐10 receptor have been associated with very early onset IBD, suggesting the essential role of homeostatic cytokines in maintaining immune homeostasis [34]. B lymphocytes may also play a role in mounting the immune response to luminal microbes through the production of secretory IgM and IgA [5]. Thus, in concert with the innate immune system, the adaptive immune system is crucial for maintaining barrier function and a healthy microbiome.

### 3.3 Microbial Dysbiosis

Gut dysbiosis, characterized by pathological changes in the gut microbial composition, has been extensively studied in patients with CD. Patients exhibit altered gut bacterial composition, with reduced representation of *Firmicutes* and *Bacteroidetes* and an overrepresentation of *enterobacteria* [35]. Studies have identified several specific taxa as potential biomarkers for disease severity and treatment response [36].

Recently, research has also focused on the role of the gut viral and fungal communities in IBD pathogenesis [37]. *Caudovirales* bacteriophage sequences and certain viral families have been associated with CD. Additionally, the fungal microbiota has been found to differ between patients with CD and healthy individuals, with an abundance of *Candida* spp. in CD patients [38]. While these associative studies highlight the presence of dysbiosis in IBD, recent publications have demonstrated that dysbiosis precedes overt gut inflammation and may be driving the development of IBD. This includes the recent longitudinal microbiome sampling of the GEM cohort, a study in first degree relatives of IBD patients [39]. This study, designed to address the issue of cause and effect, demonstrated that dysbiosis precedes clinical inflammation, suggesting it may contribute to IBD development. This temporal relationship supports dysbiosis as potential driver of IBD, not just a consequence. Additionally, the study linked dysbiosis to heightened immune activation and proinflammatory cytokines, emphasizing the interplay between the gut microbiome, genetics, and immune responses in IBD pathogenesis.

## 4 Diagnosis

Diagnosing CD requires a comprehensive approach involving medical history, physical examination, laboratory tests, imaging studies, and endoscopic evaluation as summarized in Table 1. Making the diagnosis can be challenging due to its complex and heterogenous nature, often mimicking other gastrointestinal disorders, such as UC, irritable bowel syndrome, coeliac disease and infectious gastroenteritis. Patients may present with atypical symptoms, leading to delays in diagnosis or misdiagnosis [40].

**TABLE 1:** Diagnostic and monitoring strategies.

Modality	Strengths	Weaknesses
Clinical symptoms and Symptom based scoring systems	Point‐of‐care Strong correlation with symptoms Provides reproducible score to track response	Poor specificity for diagnosis Poor correlation with endoscopic and transmural disease activity
Biomarkers	Widely available Sensitive Strong correlation with disease activity	Poor specificity
Endoscopy	Gold standard for assessment of mucosal activity Widely available	Invasive Expensive Rate of complication Requires bowel preparation
MRI	Determine transmural disease Non‐invasive No radiation Perianal evaluation Evaluates proximal small bowel	Expensive Limited by access Oral contrast required Poor colonic/rectal views Claustrophobia
CT	Widely available and accessible Non‐invasive Determine transmural disease Evaluates proximal small bowel	Expensive Oral contrast required High radiation burden
IUS	Point‐of‐care Highly acceptable to patients Non‐invasive Determine transmural disease No radiation Perianal evaluation (via perineum)	Not widely available Initial set‐up cost high Poor views of rectum, flexures and proximal SB Limited with large body habitus

### 4.1 Clinical Presentation

The clinical presentation of CD can be heterogenous and insidious. Symptoms can vary widely depending on the location of the disease, the severity of inflammation, and the behavior of the condition. Common symptoms include abdominal pain, chronic diarrhea, weight loss, fatigue, and anorexia [41]. Patients with colonic involvement may experience rectal bleeding or bloody diarrhea as the major symptoms. Approximately one‐third of patients with CD present with perianal disease, which can manifest as skin lesions (ulcerations and skin tags), anal canal lesions (stenosis, fissures, and ulcers), and fistulas with or without abscesses [5]. Additionally, high fever should always raise suspicion of a septic complication.

Overall, extraintestinal manifestations (EIMs) are present in up to 43% of patients with CD, and can affect multiple body systems, including musculoskeletal (axial and peripheral arthropathy, arthritis and ankylosing spondylitis), oral (aphthous stomatitis), ocular (uveitis, scleritis and episcleritis), dermatological (pyoderma gangrenosum, psoriasis, erythema nodosum) and hepatobiliary (primary sclerosing cholangitis) systems [42]. Some of these manifestations, such as axial arthropathy and erythema nodosum are associated with active intestinal disease, while others, such as peripheral arthropathy, pyoderma gangrenosum, are independent of disease activity [43]. Primary sclerosing cholangitis is more common in UC than in CD [43]. The prevalence of EIMs also vary by region and ethnicity, with countries throughout Asia showing consistently lower rates of EIMs than Western countries despite similar risk factors [44, 45].

### 4.2 Diagnostic and Monitoring Approaches

Diagnosing CD requires a comprehensive assessment that combines clinical history, physical examination, and complementary diagnostic tests. A thorough understanding of the patient's symptoms, family history of IBD or other immune mediated diseases, dietary habits, recent travel, gastrointestinal infections, and medication use is essential for establishing an accurate diagnosis [5]. Physical examination plays a crucial role in identifying signs of systemic toxicity, malnutrition, dehydration, anemia, or malabsorption. Perianal disease should be carefully assessed in patients with established or suspected CD.

Laboratory tests can provide additional insights. Typical findings include thrombocytosis, increased acute phase protein, especially C‐reactive protein (CRP) and anemia [5]. CRP is a surrogate serological marker of non‐specific acute inflammation and is not exclusive to intestinal inflammation. CRP has high specificity for the detection of endoscopic disease activity in patients with established diagnosis of CD [46]. However, the sensitivity is poor, and a normal CRP does not exclude the presence of active inflammation. It is estimated that about 20% of people may not mount a CRP response due to genetic single nucleotide polymorphisms in the CRP gene also affecting the sensitivity of the test [47]. Hypoalbuminaemia and vitamin deficiencies may also be present, especially in cases of extensive small bowel involvement. Although serological markers, including a positive anti‐ *Saccharomyces cerevisiae* IgA antibody (ASCA) and normal perinuclear antineutrophil cytoplasmic antibodies (pANCA), can aid in distinguishing CD from other conditions, they lack specificity for definitive diagnosis [48]. The recent PREDICTS study has shown that serum markers that were predictive of patients who will receive a diagnosis of CD within 5 years with high accuracy, including ASCA IgA and IgG, pANCA, Cbir1, OmpC, Fla2 and FlaX [49]. Fecal calprotectin (FC) is a stool biomarker where concentrations correlate with neutrophil infiltrate in the gut and represents a surrogate marker of intestinal inflammation with high sensitivity and specificity for active inflammation or organic pathology [50].

Endoscopy remains the gold standard for diagnosing CD [41]. During endoscopic procedures, such as colonoscopy and gastroscopy, the intestinal mucosa can be directly visualized. Typical endoscopic findings in CD include segmental inflammation, superficial aphthoid ulcers, and deep serpiginous ulcerations, creating a characteristic cobblestone pattern. Biopsies taken during endoscopy help confirm the diagnosis through the presence of chronic inflammation and the identification of epithelioid granulomas, although their absence does not rule out CD. Capsule endoscopy is reserved for those in whom there is high suspicion of CD despite previous negative endoscopy and radiological findings [41].

Cross‐sectional imaging tests such as CT‐enterography (CTE) or MR‐enterography (MRE) are essential for assessing the extent of disease, including transmural disease activity, and identifying complications including strictures, fistulas and abscesses [41]. MRE is preferred, when available, to reduce the risk of cumulative radiation exposure. Intestinal ultrasound is a non‐invasive and cost‐effective alternative to CTE or MRE with similar sensitivity and specificity for assessing disease activity by evaluating the intestinal wall and surrounding structures [51]. There has been a rapid uptake of IUS worldwide in CD given the benefits of high patient acceptability, strong correlation with endoscopy, low cost after initial setup, point of care results and no requirement for oral contrast [52, 53, 54].

## 5 Management and Treatment Goals

Over the past decade, the management of CD has witnessed significant changes, with improved understanding of disease progression, the development of new drugs for treatment, and improved monitoring of disease activity. Evidence has emerged that clinical factors are linked to a higher risk of progressive and disabling CD. Understanding the nature of disease progression has identified mucosal healing, which refers to the restitution of the intestinal lining and the regression or disappearance of endoscopic lesion, as an important treatment goal [55, 56]. Achieving complete remission, involving both symptomatic and endoscopic remission, is associated with improved long term outcomes. Moving towards a more stringent target of transmural healing assessed by cross‐sectional imaging techniques as well as histologic normalization is being explored as a complementary future goal [56].

### 5.1 Prognosis and Early Disease Control

Assessing the prognosis of individual patients at the time of diagnosis or during a flare is crucial for determining the appropriate therapeutic approach. Various factors associated with disease progression have been identified including younger age at diagnosis, smoking, disease duration, need for corticosteroids, presence of fistulising perianal CD, low hemoglobin and albumin levels, high CRP and calprotectin levels, and overall disease burden [57]. Stratifying patients based on these prognostic factors allows tailoring treatment strategies for high risk versus low risk individuals. Prompt diagnosis of CD is essential to prevent disease progression and complications. Diagnostic delay is common due to variable phenotypes and non‐specific clinical findings. Rapid initiation of effective treatment in patients soon after diagnosis of CD during the ‘therapeutic window of opportunity’ before bowel damage has occurred can prevent or reduce disease progression [58].

### 5.2 Treating to Target and Close Monitoring

Therapeutic goals in CD management continue to evolve. In the past, patients were typically started on aminosalicylates, steroids, or thiopurines, with escalation to more potent treatment only after these initial therapies had failed to control symptoms. However, this ‘step‐up’ approach did not effectively alter the course of the disease, leading to high rates of surgery and bowel damage. As a result, the treatment framework shifted from a focus only on symptom control to preventing disease progression, complications, and disability. Mucosal healing, defined as the absence of ulcerations on endoscopy, emerged as a significant therapeutic target as it has been shown to correlate with reduced relapse rates, decreased need for surgery, and less cumulative bowel damage [59].

Clinical symptoms alone are not always a reliable indicator of underlying inflammation, so achieving ‘deep remission’, which encompasses both clinical and endoscopic remission, has become a new treatment goal [60]. However, more research is needed through prospective disease modification trials to fully understand how these strategies will impact disease course. In the meantime, a ‘top‐down’ approach may be considered in patients with CD and poor prognostic factors, severe disease, or complicated disease [61]. This approach involves early combined immunosuppression with biologic therapies and immunomodulators and has shown slower disease progression to surgery and fewer hospital admissions for disease‐related complications [62].

For most patients, a ‘rapid step‐up’ approach is recommended, coupled with a ‘treat‐to‐target’ strategy based on close monitoring of mucosal disease and inflammation biomarkers [60]. The ‘treat‐to‐target’ strategy involves promptly adjusting treatment based on objective makers of disease activity such as CRP and FC in addition to symptom assessment [56, 63]. Using currently available biomarkers, a treat‐to‐target approach aims to achieve improved outcomes through identification of patients who require advanced drug therapy earlier in the disease course.

### 5.3 Therapeutic Agents

The choice of medication for CD depends on disease severity and response to previous therapies. Corticosteroids have long been used for induction therapy in CD due to their rapid onset of action. However, corticosteroids are not recommended for maintenance therapy due to systemic side effects and poor mucosal healing [64]. Antibiotic use should be restricted to CD complicated by fistulas or abscesses, or both [5]. Nutritional support is also a key component of managing CD, especially in patients with weight loss or malnutrition before surgery. Exclusive enteral nutrition is recommended as first‐line therapy in children with CD to induce remission [65]. Evidence for nutrition as primary therapy in adult patients is limited, however it is likely to be effective when tolerated [66].

Immunosuppressants such as thiopurines and methotrexate are generally utilized for maintenance therapy [67, 68, 69]. Thiopurines have been associated with reduced need for surgery and modest benefits in maintaining remission, but are associated with an increased risk of malignancy, especially in young men and the elderly [70, 71, 72]. However, recently their effectiveness in CD has been questioned by the results of a large study from the United Kingdom (UK) using the UK IBD bioresource that demonstrated no significant improvement with thiopurine therapy [73]. Methotrexate, while effective and commonly used in the pediatric setting, has been underutilized in adults with CD, likely due to contraindications for pregnant women and gastrointestinal side effects [68, 74].

Anti‐tumor necrosis factor (TNF) therapies, such as infliximab and adalimumab are potent agents for inducing and maintaining remission in CD [41]. These drugs are also effective in perianal disease including fistula healing [75]. However, there are concerns about immunogenicity and increased risks of infections and lymphoma in some patients, particularly when combined with thiopurines [76]. Biosimilar anti‐TNF agents have been approved for use in IBD treatment with the advantages of lower cost, making these drugs more accessible to a larger number of patients [77]. More recently developed biological drugs, such as vedolizumab and ustekinumab, are also effective in CD [78]. Vedolizumab, a monoclonal antibody targeting the α4β7 integrin, has demonstrated effectiveness in inducing and maintaining remission in luminal CD [79, 80].

Ustekinumab, targeting the shared p40 subunit of interleukin 12 and 23, has shown efficacy and effectiveness in anti‐TNF naïve and refractory CD patients including perianal disease in clinical trial and real world settings [81, 82, 83, 84, 85].

Upadacitinib, an orally available small molecule inhibits cytokine signaling pathways dependent on signal transduction and activation of transcription pathways (STAT) transcription factors selectively inhibiting Janus Kinase 1 [86], with high rates of clinical remission and a rapid onset of action and hence approval and widespread global uptake [87]. Additional oral small molecule therapies such as Etrasimod, a selective modulator of sphingosine‐1‐phosphate, have shown promise in UC but further research into CD has been terminated due to a lack of efficacy in phase 2 studies [88].

While not widely available worldwide yet, monoclonal antibodies targeting the p19 subunit of interleukin 23 (Risankizukab, Mirikizumab and Guselkumab) have shown significant promise. Risankizumab has shown efficacy in phase III ADVANCE, MOTIVATE and FORTIFY trials, with high rates of clinical remission after 12 weeks of therapy [89]. Mirikizumab has shown safety and efficacy in the phase III VIVID‐1 trial, with high rates of clinical and endoscopic remission to 52 weeks of therapy, with similar rates of adverse events to ustekinumab [90]. Guselkumab shows efficacy in CD with high levels of clinical remission and endoscopic response at both week 12 and 48 in the GRAVITI phase 3 study [91].

The choice of biologic therapy depends on patient characteristics, comorbidities, efficacy, safety, and the route of administration (as summarized in Table 2).

**TABLE 2:** Advanced drug therapies for Crohn's disease.

Advanced drug therapy	Mechanism of action	Route of administration	Biosimilar available
Infliximab	Anti‐tumor necrosis alpha inhibition	Induction—intravenous Maintenance—intravenous or subcutaneous	Intravenous—Yes Subcutaneous—No
Adalimumab	Anti‐tumor necrosis alpha inhibition	Induction and maintenance—subcutaneous	Yes
Ustekinumab	P40 subunit of Interleukin 12 and 23 inhibition	Induction—intravenous Maintenance—subcutaneous	Yes
Vedolizumab	Alpha‐4 beta‐7 integrin inhibition	Induction—intravenous Maintenance—intravenous or Subcutaneous	No
Upadacitinib	Janus Kinase Inhibition	Oral	No
Guselkumab	p19 subunit of Interleukin 23 inhibition	Induction—intravenous or Subcutaneous Maintenance—subcutaneous	No
Mirikizumab	p19 subunit of Interleukin 23 inhibition	Induction—intravenous Maintenance—subcutaneous	No
Risankizumab	p19 subunit of Interleukin 23 inhibition	Induction—Intravenous Maintenance—subcutaneous	No

### 5.4 Role of Surgery and Endoscopic Intervention

Surgery has traditionally been considered for patients with refractory medical disease, complications (strictures with or without internal fistulisation, abscesses or malignancy), intolerance to medical therapy, or obstructive symptoms [1]. In stricturing CD, conventional therapy alone has been shown to improve symptoms and stricture morphology, with dose escalation compounding this effect [92]. The LIR!C trial suggested an alternative approach, showing that upfront laparoscopic ileocolonic resection is a cost‐effective treatment with similar quality of life outcomes to treatment with anti‐TNF therapies in patients with CD with limited (affected segment ≤ 40 cm) and predominantly inflammatory terminal ileitis for whom conventional treatment is not successful [93]. In comparison, the use of endoscopic balloon dilatation in combination with advanced drug therapies has been shown to be technically successful, to reduce need for further surgical procedures and appears to be cost effective [94, 95, 96]. Multidisciplinary discussion and appropriate pre‐operative evaluations are crucial before deciding on endoscopic or surgical management. Advances in minimally invasive surgery have led to shorter hospital stays, faster recovery times, and improved cosmetic outcomes for CD patients undergoing surgical intervention [97].

### 5.5 Fertility and Pregnancy

Fertility rates for CD patients in remission without a history of pelvic surgery are similar to those of the general population. Active disease during pregnancy may be associated with adverse pregnancy outcomes, such as preterm birth and stillbirth [98]. Reassuringly, most IBD medications, except methotrexate and JAK inhibitors, are considered safe during pregnancy and breastfeeding [99]. The mode of delivery should be determined by obstetric indications, and careful management of medication is essential for pregnant patients with CD. Perianal CD or active proctitis are situations in which a Caesarean section may be preferred.

### 5.6 Dietary Therapy

Diet plays a vital role in gut microbial biodiversity and health in general, along with a significant role in the induction and maintenance of remission in CD. The Crohn's Disease Exclusion Diet (CDED), which involves exclusion of certain foods (such as gluten, dairy, emulsifiers, alcohol and coffee to name a few), often combined with partial enteral nutrition, has proven effective in induction and maintenance of clinical remission in mild–moderate Crohn's Disease, and may lead to endoscopic remission [100, 101, 102].

Exclusive Enteral Nutrition (EEN) has been used in pediatric CD for over 20 years, with rates of induction of remission comparable to oral corticosteroid [103]. While evidence is less clear in an adult population, benefit is seen with a short‐course of therapy either as an adjuvant treatment to therapeutic switch or in place of traditional corticosteroid [104, 105]. One challenge encountered more commonly when using EEN in an adult population is lower rates of adherence and tolerance to EEN given the enforced avoidance of all solids [106].

Dietary therapy has a role in induction and maintenance of remission in Crohn's Disease, with best evidence for CDED and EEN, although adherence and tolerance rates are variable.

### 5.7 General Health Maintenance and Follow‐Up

Regular follow‐up is essential for CD patients due to the risk of disease flare‐ups and long‐term complications. Smoking cessation is strongly encouraged, and patients should receive guidance on vaccinations, osteoporosis screening, and cancer or dysplasia surveillance. Quality of life is a crucial aspect of CD management. The chronic progressive nature of the disease can negatively impact a patient's social, educational, professional, and family activities. Factors such as ongoing disease activity, the presence of EIMs, and medication use can affect patient's health‐related quality of life, including emotional and psychological issues such as fatigue, anxiety and depression, social and family interactions, and work productivity [107, 108]. Screening for early signs of depression or anxiety and the initiation of pharmacologic or psychological treatment when appropriate can lead to improved functioning and positively impact the course of disease. Measuring and addressing quality of life is essential in understanding patients' perception of their health and tailoring treatment approaches to improve quality of life. A number of validated tools for assessing patient reported health outcomes, such as IBD Disk, have been shown to accurately predict daily life burden and parallel CD activity, allowing for an objective measure of disease burden that can be carried forward and assessed longitudinally [109, 110].

## 6 Outlook

CD is a chronic and debilitating inflammatory disorder that affects the gastrointestinal tract. In the past two decades our understanding of CD has evolved, and it is now recognized as a progressive disease involving destructive inflammation resulting in cumulative damage that can lead to severe complications. As the incidence of CD increases worldwide, researchers and healthcare professionals are focused on developing therapeutic strategies aimed at achieving mucosal healing and preventing disease progression.

### 6.1 Disease Progression and Prognostic Factors

Approximately 50% of CD patients experience disease progression within 10 years of diagnosis [1]. To assess bowel damage and stratify patients for risk of disease progression the Lémann score was developed. The Lémann score is a promising tool that measures cumulative structural damage to the bowel through endoscopic assessment of strictures, penetrating lesions, and the need for surgical resection [111]. By identifying high‐risk patients for rapid disease progression, the Lémann score helps inform clinical decisions on modifying therapy. Additionally, the international Organization for the Study of Inflammatory Bowel Diseases has created an index to define overall disease severity based on the presence of mucosal lesions, a history of a fistula, a history of an abscess and a history of intestinal resection [60]. Bowel damage and the Lémann score have been identified as independent prognostic factors for the requirement of intestinal surgery and CD‐related hospitalization during patient follow‐up [111].

Preventing disease progression has become a primary goal in CD management. Research is ongoing to identify predictive biomarkers and develop toolkits to measure disease severity and progression accurately [112]. The incorporation of personalized medicine in CD treatment is also gaining momentum, aiming to customize healthcare based on individual genomic, clinical, and environmental information [113]. Models to predict disease outcomes, such as a validated web‐based predictive tool that considers various clinical and molecular factors, are being developed for early intervention and improved disease management [114].

### 6.2 Treatment Approaches and Emerging Therapies

Currently available treatments for CD have limitations and new therapeutic options are needed. Targeted biologic therapies have been a significant advance in CD treatment but have generally required a parenteral route of administration and potential immunogenicity. Further orally administered small molecules including selective Janus kinase (JAK) inhibitors and sphingosine‐1‐phosphate receptor 1 agonists are emerging as potential therapies [115, 116]. Early phase trials into the inhibition of TNF‐like cytokine 1A (TL1A), OX40 ligand, receptor interacting protein kinase 2 (RIPK2), and C—C chemokine receptor 9 (CCR9) have also show promise [117, 118, 119, 120]. Antibiotic regimens and microbiota manipulation through diet or fecal microbiota transplantations are also under investigation as potential treatment options. Physicians must consider many factors during the decision‐making process including comparative effectiveness and long‐term safety profile, product availability and labelling, adherence to international guidelines, cost considerations, and the preferences of patients [121].

The development of personalized medicine in CD treatment involves not only considering symptoms, serological markers, and endoscopic and histological assessment but also genetic and molecular phenotypes. Optimization through therapeutic drug monitoring holds promise for individualized dose adjustment, considering correlations between anti‐TNF trough concentrations, anti‐drug antibodies, and disease outcomes [122, 123]. Although most trials have not demonstrated superior results for proactive trough‐level‐based dose intensification disease activity‐based doss escalation, therapeutic drug monitoring remains valuable for assessing treatment response and guiding therapeutic choices [122].

In response to cost pressures and concern for side‐effects of medical therapy, de‐escalation is also being explored in CD management. Ceasing immunosuppressant monotherapy after a period of remission has been studied, but it is associated with increased rates of relapse [124]. Ceasing immunosuppressants in patients on combination therapy with immunosuppressants and anti‐TNF agents appears to have more favorable outcomes in terms of relapse rates [125]. Further de‐escalation strategies are being investigated, and ongoing studies are exploring the concept of cycling therapy to reduce costs and toxicity [126].

### 6.3 Future Directions

Future directions for CD management encompass various aspects, including predictive biomarkers, therapeutic sequencing algorithms, and ongoing research to improve treatment outcomes.

One area of ongoing investigations is the prediction of treatment response. Researchers are exploring the gut microbiome as a potential predictor of response to currently available biologic therapies [127, 128, 129]. Certain gut microbial signatures have been associated with treatment responses. Additionally, genomic and proteomic markers are being studied to predict how CD patients may response to anti‐TNF therapies [130, 131, 132, 133]. Biomarker‐driven selection of therapies and the consideration of combination therapies, like vedolizumab or anti IL‐23 with a JAK inhibitor or anti‐TNF agent, are being explored as future strategies to improve treatment outcomes. Initial data from real‐world studies have shown promise and safety in using combination biologic therapy [134, 135, 136, 137, 138].

As new advanced therapies continue to be developed for CD treatment, choosing the most appropriate agent may become challenging. Treatment algorithms for CD are expected to evolve with the introduction of new drugs and the increasing use of biosimilars. Comparative head‐to‐head trials and studies that combine drugs targeting different pathways will be crucial in shaping the therapeutic landscape and prognosis of CD in the future.

Another challenging area in CD management is the prevention of the disease. Currently, there are no established antigenic drivers or robust vaccines to prevent CD [139]. While researchers have explored potential preventative interventions, such as gut microbial manipulations, the goal of preventing CD remains elusive.

## 7 Conclusion

CD is a complex and challenging inflammatory disorder that demands ongoing research and therapeutic advancements. The concept of progressive disease has driven the focus towards preventing disability and complications globally. Personalized medicine and drug monitoring hold significant promise in optimizing CD treatment and individualizing therapy for each patient. The emergence of orally administered small molecules and selective biologic therapies provides new hope for improved outcomes in CD management. However, the pathogenesis and prevention of CD remain areas that require further investigation. Future research should focus on predictive biomarkers and treatment algorithms, enabling early intervention and better disease management. As the understanding of CD continues to evolve, the future holds the promise of more effective and targeted therapies for this debilitating disease.

## Funding

The authors have nothing to report.

## Conflicts of Interest

Yoon‐Kyo An reports grants from Janssen, during the conduct of the study; has received speaking and consulting fees from Abbvie, Bristol Myers Squibb, Celltrion, Chiesi, Dr. Falk, Ferring, Janssen, Pfizer, Sandoz, Shire and Takeda; served on advisory boards member for Abbvie, Bristol Myers Squibb, Chiesi, Janssen, NPS Medicine wise, Microba; received research and educational funding from Abbvie, Celltrion, Dr. Falk, Janssen, Pfizer, Sandoz and Takeda. Graham Radford‐Smith has served on advisory boards for Janssen, Novartis, Takeda, Ferring, Abbvie, Organon, GSK, and Pfizer, has received research funding from Janssen, and has received speaker's fees from Janssen, Takeda, Pfizer, Ferring, Novartis, and GSK. Jakob Begun has received speaking and consulting fees from Abbvie, Bristol Myers Squibb, Celltrion, Chiesi, Dr. Falk, Ferring, Janssen, Pfizer, Sandoz, Shire, and Takeda; served on advisory boards member for Abbvie, Bristol Myers Squibb, Chiesi, Janssen, NPS Medicine wise, Anatara, Microba; received research and educational funding from Abbvie, Janssen, Pfizer, and Takeda. Robert Gilmore has no conflicts to disclose.

## Data Availability

Data sharing not applicable to this article as no datasets were generated or analyzed during the current study.
